# Acylation of Antioxidant of Bamboo Leaves with Fatty Acids by Lipase and the Acylated Derivatives’ Efficiency in the Inhibition of Acrylamide Formation in Fried Potato Crisps

**DOI:** 10.1371/journal.pone.0130680

**Published:** 2015-06-22

**Authors:** Xiang Ma, Erpei Wang, Yuyun Lu, Yong Wang, Shiyi Ou, Rian Yan

**Affiliations:** Department of Food Science and Engineering, College of Science and Engineering, Jinan University, Guangzhou, 510632, China; University of Tsukuba, JAPAN

## Abstract

This study selectively acylated the primary hydroxyl groups on flavonoids in antioxidant of bamboo leaves (AOB) using lauric acid with *Candida antarctica* lipase B in *tert*-amyl-alcohol. The separation and isolation of acylated derivatives were performed using silica gel column chromatography with a mixture of dichloromethane/diethyl ether/methanol as eluents. Both thin layer chromatography and high-performance liquid chromatography analyses confirmed the high efficiency of the isolation process with the purified orientin-6″-laurate, isoorientin-6″-laurate, vitexin-6″-laurate, and isovitexin-6″-laurate that were obtained. The addition of AOB and acylated AOB reduced acrylamide formation in fried potato crisps. Results showed that 0.05% AOB and 0.05% and 0.1% acylated AOB groups significantly (*p* < 0.05) reduced the content of acrylamide in potato crisps by 30.7%, 44.5%, and 46.9%, respectively.

## Introduction

For decades, there has been a focus on secondary plant metabolites due to their potential antioxidant properties and widespread usage as natural additives in the food, pharmaceutical and cosmetic industries. Flavonoids, which are plant polyphenol secondary metabolites, are found in fruits, vegetables, and herbs, and due to their effects on human health, such as anti-cancer and anti-cardiovascular activities, they have been researched and reported on intensely [1,2]. In addition, other properties of flavonoids including anti-allergenic, anti-viral, anti-microbial, anti-mutagenic and anti-inflammatory activities have also been the focus of much research [3,4,5]. Generally, due to the hydrophilicity of glycosylated flavonoids, their instability and insolubility in organic media still hinder their application [6,7]. However, it has been hypothesized that by improving their solubility in hydrophobic environments, the drawbacks of glycosylated flavonoids could be easily overcome [8,9]. Chemical and enzymatic acylation have been investigated, but the enzymatic method is more promising. Compared with chemical acylation, the enzymatic approach is more favorable in terms of acylation for enzyme regioselectivity, and the acylation is conducted under milder conditions. Researchers have utilized *Candida antarctica* lipase B (CALB) as a biocatalyst and found that the acylation mainly occurs at the primary hydroxyl groups on the glucose portion of the glycosylated flavonoids [10,11,12,13].

**Fig 1:**
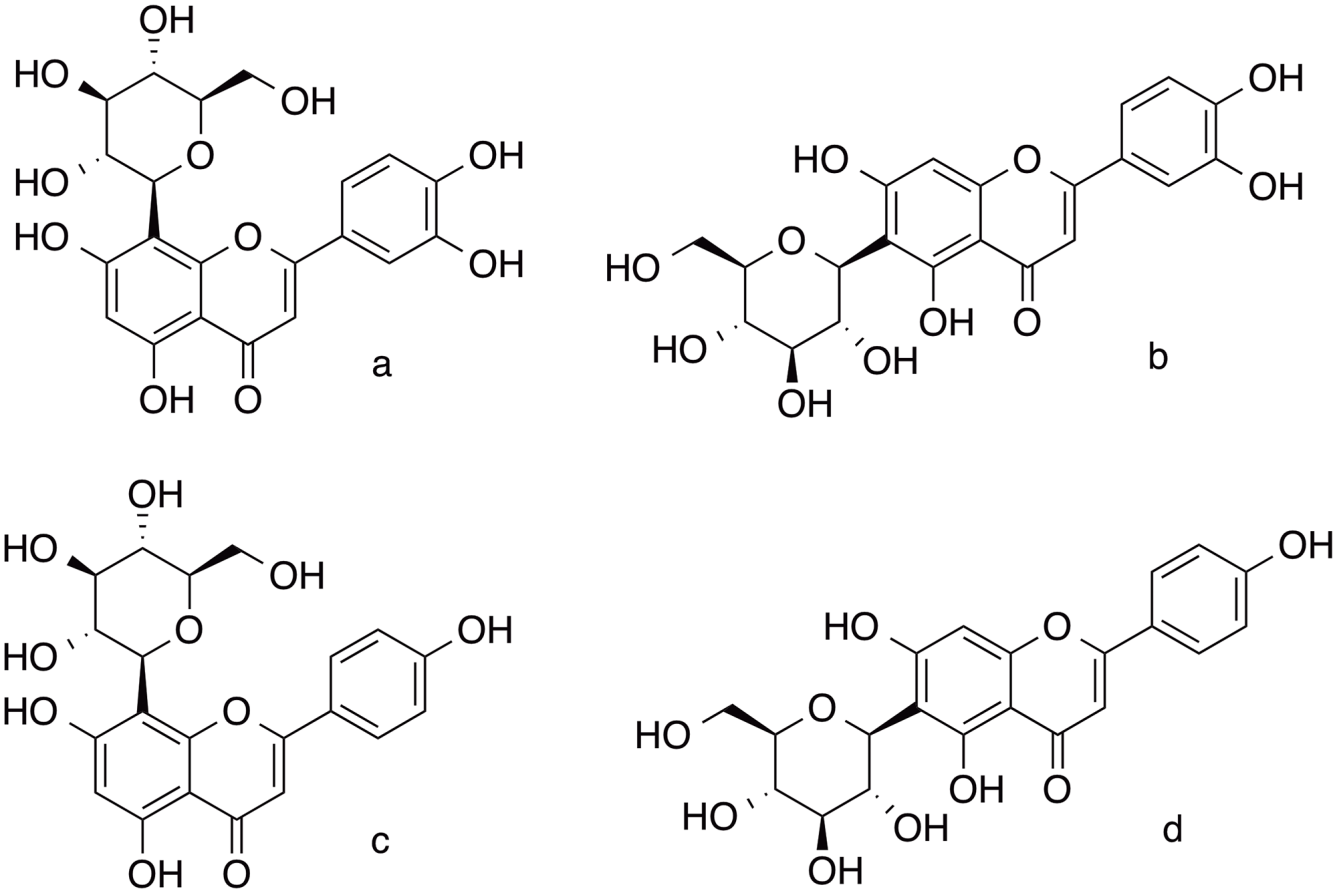
Structures of flavonoids in AOB. (a) orientin, (b) isoorientin, (c) vitexin, and (d) isovitexin.

In China, the Ministry of Health approved antioxidant of bamboo leaves (AOB) as a natural food additive in 2007. AOB can be used as a food antioxidant, preservative, or flavoring in many types of foods. AOB has several types of bioactive components such as flavonoids, lactones, and phenolic acids, but it consists mainly of four representative flavonoids (orientin, isoorientin, vitexin, and isovitexin) (Fig 1) [14]. Since Swedish scientists reported the acrylamide formation pathway and mechanism in 2002 [15], many researchers have shown interest in this topic, and considerable research resources have been invested into the reduction of acrylamide in foods. In particular, fried and baked carbohydrate-rich foods are extensively studied because Maillard reactions (which are suspected to be a source of acrylamide) are more prone to occur in these types of foods during processing [16,17]. Chinese researchers have also studied AOB’s inhibition of acrylamide formation, and it has been reported that AOB can effectively reduce acrylamide formation in model reactions and food systems. The premium reduction rate of acrylamide in an asparagine-glucose microwave heating model system was 74.4% when the level of AOB added was 10^−4^ mg mL^-1^, and nearly 74.1% of acrylamide in potato crisps was reduced when the AOB addition level was 0.1% (w/w) [18,19].

Although there has been much research regarding the acylation of different types of flavonoids, the direct acylation of plant extracts rich in flavonoids is rarely reported, and there are few reports regarding the acylated flavonoid inhibition of acrylamide formation in fried foods. Therefore, this study focuses on the enzymatic acylation of AOB by CALB with lauric acid as acyl donors and on efficient approaches to separate and isolate the flavonoid esters in acylated products. The second section evaluates and compares the derivatives’ antioxidant activities and efficiencies in the reduction of acrylamide in fried potato crisps.

## Materials and Methods

### Enzymatic acylation of AOB

The AOB was supplied by Golden Plate Bio-Tech Co., Ltd., Hunan, China. The manufacturer stated that the antioxidant consisted of 80% (w/w) flavonoids with 13% orientin, 39% isoorientin, 5% vitexin, and 22% isovitexin. Enzymatic acylation of AOB was conducted through enzymatic esterification of flavonoids with lauric acids (2.5 g AOB (containing 4.5 mM flavonoids, 1 eq) and 4.51 g lauric acid (22.5 mM, 5 eq). In a 250 mL round bottom flask, 100 mL anhydrous t*ert*-amyl-alcohol AOB and lauric acids were combined under nitrogen. After the complete dissolution of the substrates, the acylation was initiated by adding 10.0 g Novozyme 435 (immobilized CALB, Novozyme, Tianjing, China) at 65°C, and the reaction was maintained at this temperature for 48 h. The water content of the reaction medium was maintained at less than 0.1 water activity with the addition of 100 mg mL ^-1^ 4 Å molecular sieves (supplied by Sinopharm Chemical Reagent Co., Ltd., Shanghai, China) [20]. The enzyme and molecular sieves were then filtered off, and the filtrate was concentrated. The residue was dissolved with 100 mL ethyl acetate and extracted with 2 × 100 mL water. The organic layers were concentrated again, dissolved in 100 mL acetonitrile, and extracted by 2 × 100 mL *n*-hexane. The acetonitrile layer was then filtered through a pan of silica gel 60 (Qingdao Haiyang Chemical Co., Qingdao, China) and washed with 50 mL ethyl acetate. The combined organic layers were washed with 100 mL brine, dried by sodium sulfate, concentrated and placed on a silica gel column, and eluted by a solvent mixture of dichloromethane/diethyl ether/methanol at a ratio of 80:15:5, v/v/v. Preparative high-performance liquid chromatography (HPLC) was performed on an Agilent 1260 infinity preparative chromatography system equipped with an Agilent Prep LC controller, a G1365B MWD detector (Agilent, Santa Clara, CA) and a Bondapak C18 semi-preparative column (I.D. 300 × 7.8 mm, 10 μm, Waters, Milford, MA). A linear gradient of HPLC-grade acetonitrile/water (both containing 0.1% HPLC-grade formic acid, v/v) with a flow rate of 4 mL min ^-1^ for 0 min (10:90, v/v), 50 min (100:0, v/v), and 60 min (10:90, v/v) was selected. The ejection volume was 2 mL. The elution was conducted at room temperature (*approx*. 22°C) at 280 nm with an ultraviolet detector (UVD). All of the HPLC-grade solvents were supplied by J.T. Baker, USA, and the other solvents were AR-grade and supplied locally.

### Analyses of acylated derivatives

HPLC and thin-layer chromatography (TLC) were used in the analyses of the acylated derivatives. HPLC analyses were conducted with an Essentia LGE UV system (Shimadzu, Kyoto, Japan) equipped with a column (Syncronis C18, 250 × 4.6 mm, Thermo, Guangzhou, China) and an UVD (SPD-10A, Shimadzu, Kyoto, Japan). For all samples, a linear gradient of acetonitrile/water (both containing 0.1% formic acid, v/v) for 0 min (10:90, v/v), 30 min (100:0, v/v), and 35 min (10:90, v/v) was used. The injection volume was 20 μL, and the flow rate was 1 mL min ^-1^. The elution was conducted at room temperature (*ca*. 22°C) at 280 nm with the UVD [21]. The TLC analyses were performed on glass-backed 0.2-mm-thick silica gel 60 F254 plates (supplied by Qingdao Haiyang Chemical Co., Qingdao, China) with a solvent mixture of dichloromethane/diethyl ether/methanol (65:30:5, v/v/v). Visualization of the eluted plates was performed under a 254 nm UV lamp. Proton (^1^H) NMR spectra of orientin-6″-laurate and vitexin-6″-laurate were recorded at room temperature in base-filtered DMSO-*d*6 on a Bruker AC500 spectrometer operating at 500 MHz for proton nuclei, and the ^1^H NMR data of isoorientin-6″-laurate and isovitexin-6″-laurate were reported previously [21].

Orientin-6″-laurate: ESI-MS: 629.2 [M-H]^-^, 611.2 [M-H_3_O]^-^; [α]_D_^20^ +15.6(*c* 1.0, methonal); ^1^H: *δ* (ppm) 13.21 (s, 1H, OH5), 7.41 (s, 1H, H2′), 7.41−7.36 (m, 1H, 6′), 6.89 (s, 1H, H5′), 6.61 (d, 1H, H3), 6.31 (s, 1H, H6), 4.60 (d, 1H, H1″), 4.10 (m, 1H, H4), 3.92 (dd, 2H, H6″), 3.35 (m, 1H, H5″), 3.21 (d, 1H, H3″), 3.18 (m, 1H, H4″), 2.51 (m, 2H, CH2α fatty chain), 1.41 (dd, 2H, CH2β fatty chain), 1.20 (d, 16H, CH2 fatty chain), and 0.85 (d, 3H, CH3 fatty chain).

Vitexin-6″-laurate: ESI-MS: 613.1 [M-H]^-^, 595.1 [M-H_3_O]^-^; [α]_D_^20^–17.0(*c* 1.0, methonal);^1^H: *δ* (ppm) 13.19 (s, 1H, OH5), 7.98 (s, 2H, H2′, H6′), 6.94 (s, 2H, H3′, H5′), 6.81 (s, 1H, H3), 6.28 (s, 1H, H6), 4.72 (m, 1H, H1″), 4.10 (s, 1H, H2″), 3.91 (m, 2H, H6″), 3.55 (s, 1H, H3″), 3.34 (s, 1H, H5″), 3.18 (s, 1H, H4″), 2.51 (s, 2H, CH2α fatty chain), 1.40 (s, 2H, CH2β fatty chain), 1.26−1.21 (m, 16H, CH2 fatty chain), and 0.82 (s, 3H, CH3 fatty chain).

### Preparation of potato crisps

The potato crisps were prepared according to the method of Zhang *et al*. [19]. Fresh potato tubers were washed, peeled and sliced evenly to a thickness of approximately (1.2 ± 0.2) mm. The slices were washed twice by hand with water and dried using adsorbent paper. Then, the slices were divided into different test groups and immersed for 60 s in hydrous ethanol (ethanol:water = 95:5, v/v) solutions containing 0.001%, 0.01%, 0.05%, 0.1%, 0.5%, or 1% (w/w) AOB and acylated AOB. Slices in the control group were immersed in only hydrous ethanol. Following treatment, the slices were dried in an oven at 50°C for approximately 30 min. Next, both the control group and test groups ((55 ± 5) g per batch) were placed in a large basket in an 8 L electric frying pan (Baote, Wuhan, China) with palm oil. To ensure that the slices were not over-cooked, slices were carefully added so that they could move freely. All potato slices were immersed in the palm oil for exactly 4 min at 170°C. After cooking, all potato crisps were submitted to a quantitative analysis of acrylamide.

### Sample pretreatment and acrylamide quantification

The standards (provided by Sigma-Aldrich) contained concentrations of acrylamide ranging from 0 to 10 μg mL ^-1^ (0.0, 0.5, 1.0, 2.0, 4.0, 6.0, 8.0, and 10.0). Analyses of these samples was conducted on a Shimadzu LC-20AT system (Shimadzu, Kyoto, Japan) consisting of an LC-10ATvp pump, a SIL-HT autosampler, and a CTO-10Asvp temperature-controlled column oven. These samples were eluted on a Zorbax SB-AqC18 column, (250 mm × 4.6 mm, 5 μm) with a mobile phase of water/methanol (95/5, v/v) at a flow rate of 0.5 mL min ^-1^. The injection volume was 5 μL, and the column temperature was maintained at 30°C. Acrylamide was quantified at 204 nm using the photo diode array detectors. The equation of linear regression for the calibration curve was *y* = *113144x−1103*.*4* with a relation coefficient (r^2^) of 0.9995.

Potato crisp samples were defatted using petroleum ether with a boiling-point range of 40–60°C. The combined organic layers were then extracted with a 2 mol L^-1^ aqueous solution of sodium chloride, and the organic layers were discarded. The combined aqueous layers were then extracted with ethyl acetate before being purified by solid-phase microextraction (SPME). All the SPME cartridges (Welchrom C18E, 6 mL, 500 mg, Welch Materials Inc., Maryland, MA) were conditioned with 5 mL of methanol followed by 5 mL of water. The methanol and water portions were discarded. Each cartridge was loaded with 1.5 mL of dissolved extract. The extract was allowed to pass through the sorbent material and discarded. Then, the cartridge was eluted with 3 mL of water, which was collected and analyzed. The concentration ratios of acrylamide in samples were determined from measured response ratios and calculated using the linear regression equation. Acrylamide concentration means are presented with standard deviations. This experiment was performed in triplicate. The SPSS 13.0 statistical package (SPSS, Inc., Chicago, IL) was used to perform the statistical analyses. Paired samples *t* test was applied to determine whether a particular treatment of the sample would result in significantly different content of acrylamide compared with the control. Treatment differences with *p* < 0.05 were considered to be significantly different.

## Results and Discussion

### Production of AOB acylated derivatives

**Fig 2:**
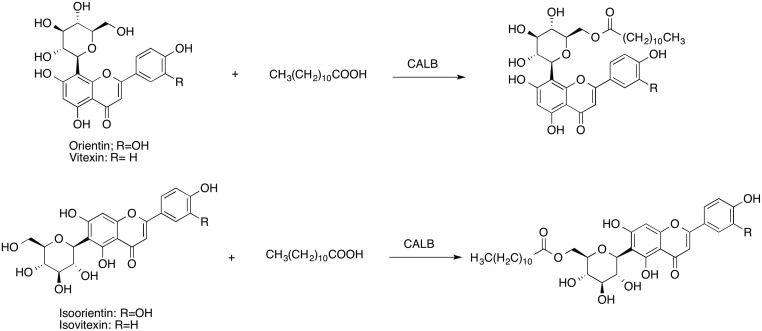
Acylation of orientin, virexin, isoorientin, and isovitexin with CALB.

Fig 2 shows the acylation of orientin, virexin, isoorientin, and isovitexin with lauric acids. All four flavonoids have a glucose moiety, and acylation occurred at the 6″-OH on the glucose moiety as a result of the high reactivity of the primary hydroxyl groups and the good selectivity of CALB. Ardhaoui *et al*. [22] and Gao *et al*. [23] also reported similar results for the acylation of other glycoside flavonoids and showed that with CALB, acylation occurs at the primary hydroxyl group on the glucose. Solvents and acyl donors are the two major concerns during this type of biosynthesis reaction. Gayot *et al*. [24] and Xu *et al*. [25] found that *tert*-amyl-alcohol was the most suitable solvent for the acylation of flavonoids with fatty acids due to *tert*-amyl-alcohol’s ideal log *P* (1.15), which is less prone to destroy the enzymes’ stabilization water layers. In contrast, other solvent candidates such as *tert*-butanol and acetone have log *P* values of 0.8 and −0.23, respectively, which have negative effects on the stability of enzymes [26]. In terms of the acyl donors, Ardhaoui *et al*. [22] found that for fatty acids with a carbon chain shorter than 12, both acylation yields and initial reaction rates increased progressively to peaks at 76% and 3.5 × 10^−3^ mM h ^-1^ with lauric acid (C12). For fatty acids with longer carbon chains (C14-C18), no significant difference was observed. Mellou *et al*. [27] also found that when conducting acylation through transesterification using various vinyl fatty acid esters, the reaction rate and yields with vinyl laurate (C12) were higher than with vinyl decanoate (C10) and vinyl stearate (C18).

### Purification and isolation of AOB acylated derivatives

**Fig 3:**
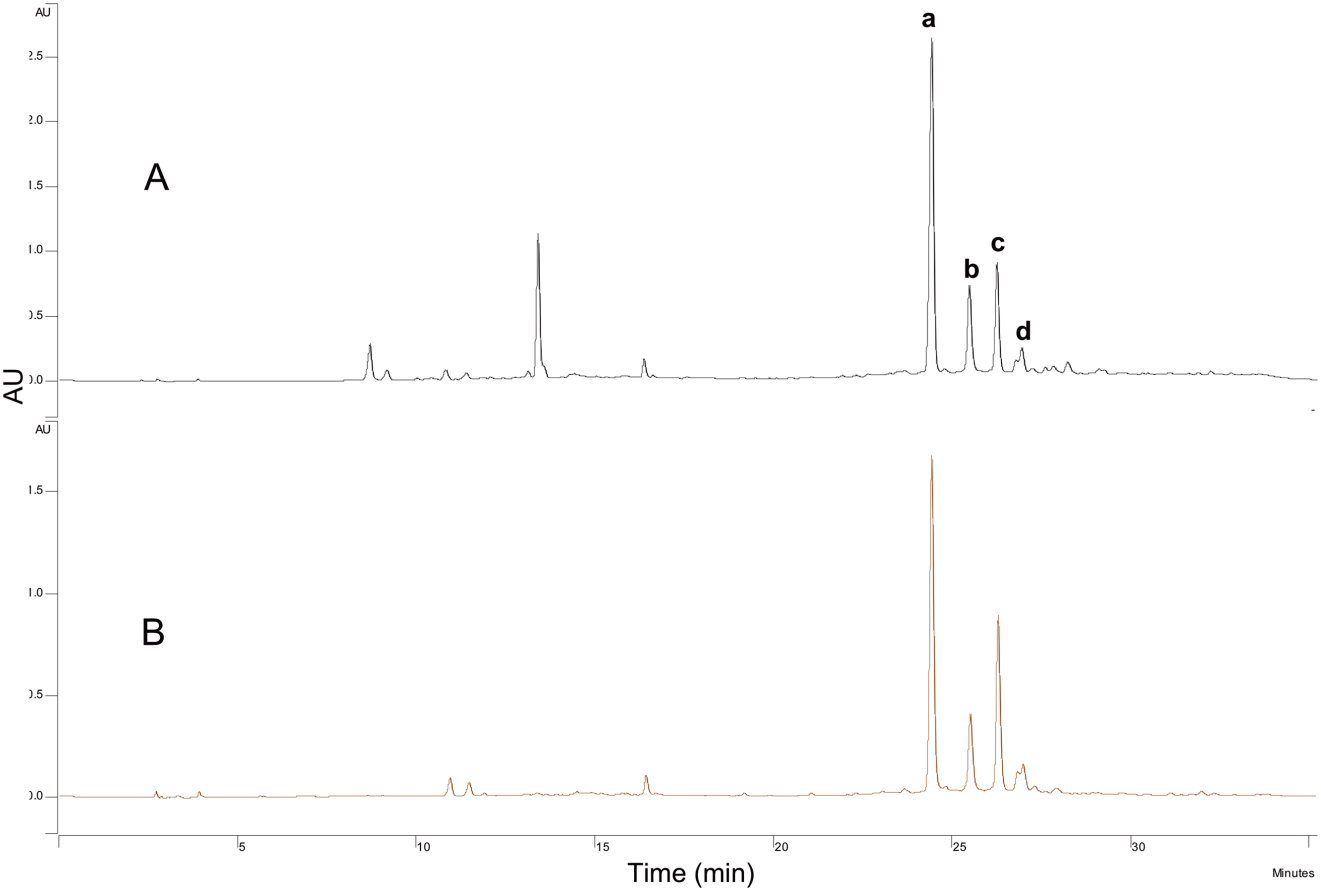
HPLC profile of AOB acylated derivatives mixture. A: mixture obtained after liquid/liquid extraction; B: further purification by filtration through silica gel. (a: isoorientin-6″-laurate; b: orientin-6″-laurate; c: isovitexin-6″-laurate; d: vitexin-6″-laurate)

**Fig 4:**
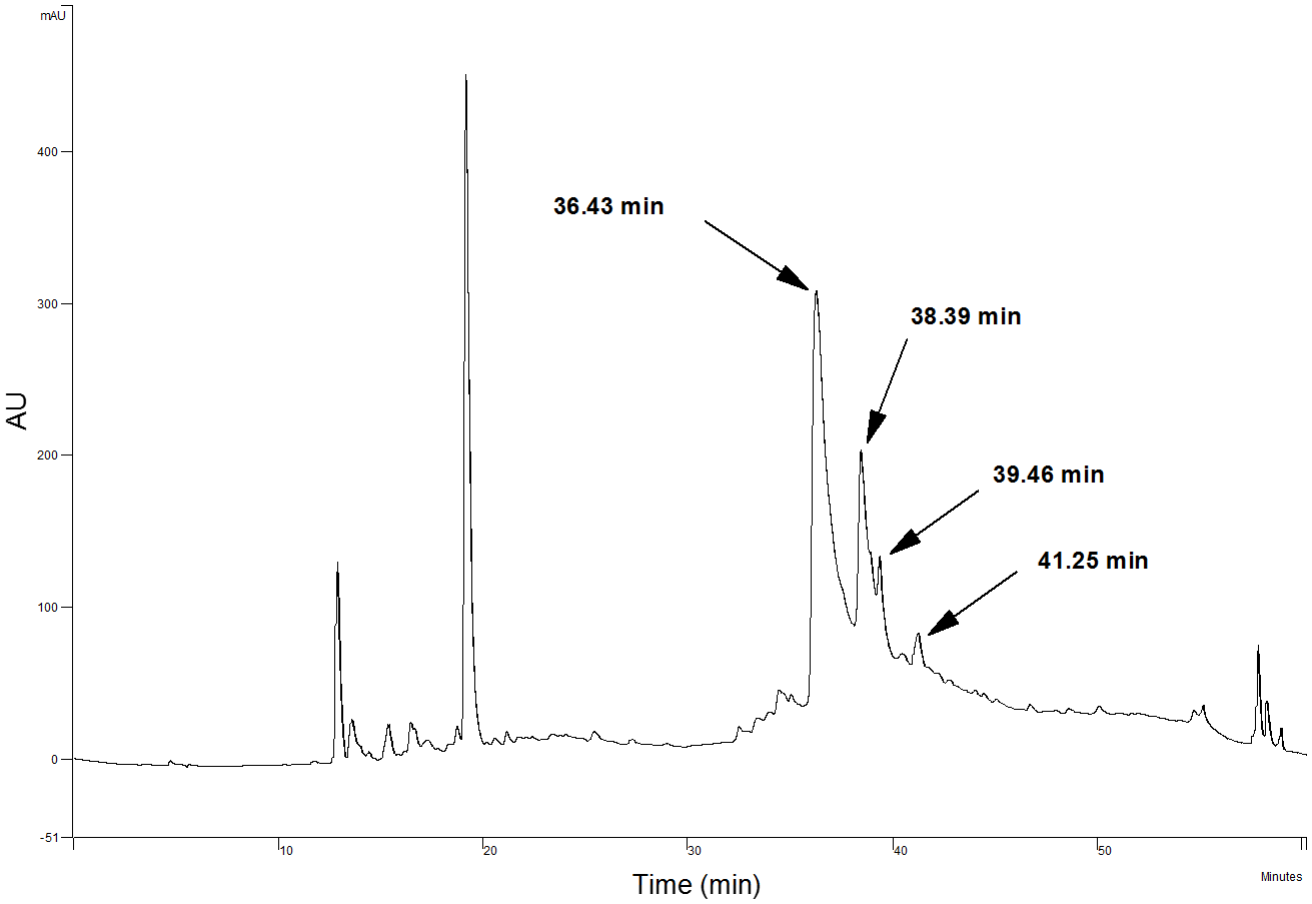
Preparative HPLC profile of AOB acylated derivative mixture.

**Fig 5:**
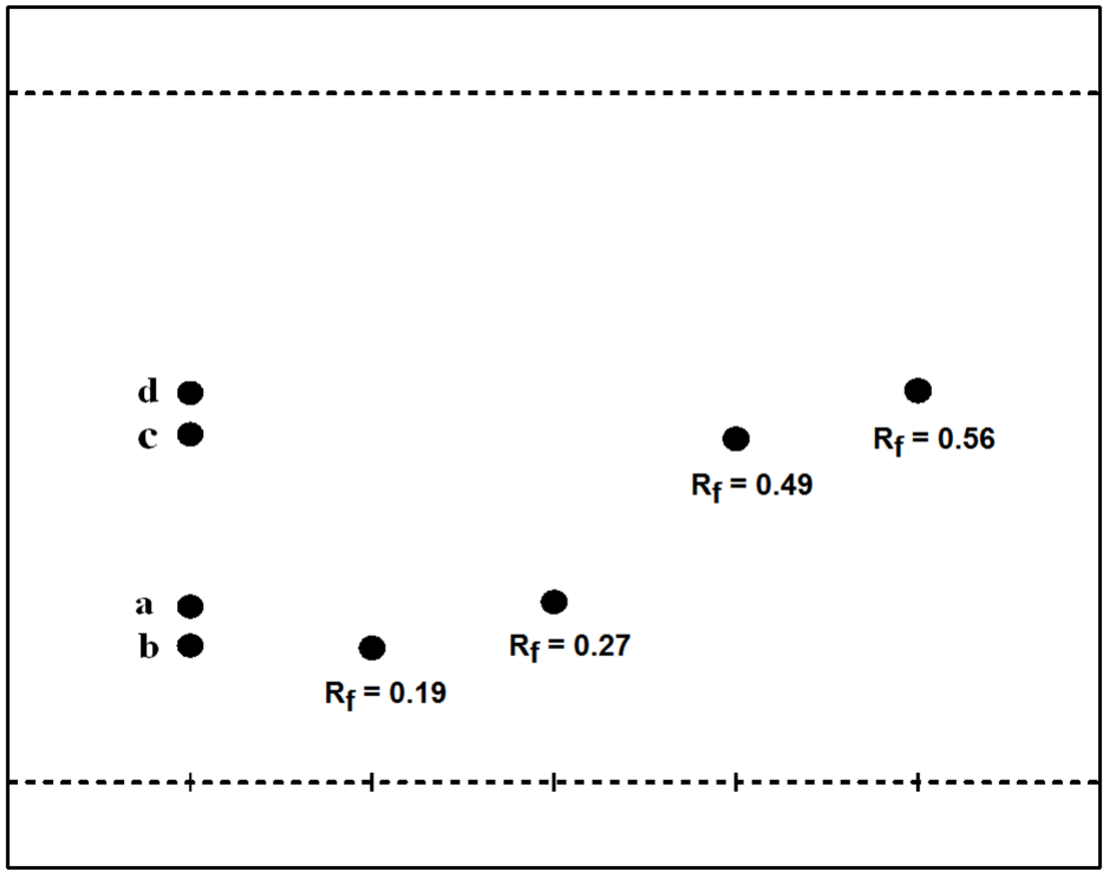
TLC profile of separated products. (a: isoorientin-6″-laurate; b: orientin-6″-laurate; c: isovitexin-6″-laurate; d: vitexin-6″-laurate. Solvenr for elution: dichloromethane/diethyl ether/methanol(80:15:5, v/v/v))

**Fig 6:**
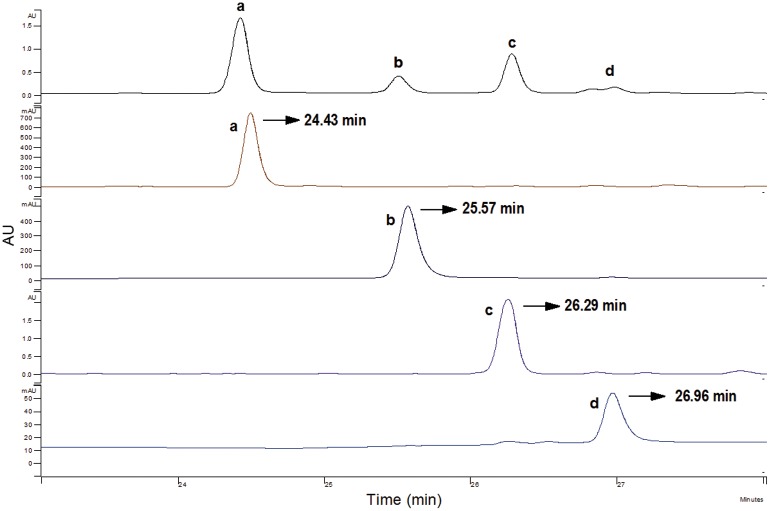
HPLC profile of separated products. (a: isoorientin-6″-laurate; b: orientin-6″-laurate; c: isovitexin-6″-laurate; d: vitexin-6″-laurate)

HPLC analyses confirmed that the liquid/liquid extraction step was not sufficient to separate AOB or the acylated derivatives, as both components were found at high levels (Fig 3A). Fig 3B shows that the content of residual flavonoids was much lower after filtration through silica gel. Previous studies have discussed the preparative isolation of orientin, isoorientin, vitexin, and isoorientin from plant extracts using the preparative HPLC method. For example, Zhang *et al*. established the simultaneous purification and isolation of flavonoids in bamboo leaf extracts using AB-8 resin-based column chromatography followed by preparative HPLC using a mobile phase consisting of 10% and 15% (v/v) of acetonitrile and 1% acetic acid in water [14]. Similarly, the separation and isolation of flavonoid esters can be performed on preparative HPLC. Ardhaoui *et al*. performed the separation of quercetin, hesperidin, rutin and esculin fatty acids esters directly through preparative HPLC using a methanol/acetic acid solution (water/acetic acid, 97:3, v/v)(at 0 min (30:70, v/v), 5 min (100:0, v:v), 10 min (100:0, v:v), 15 min (30:70, v/v)) [22]. In this study, the possibility of using the preparative HPLC method were investigated, but it was not sufficient to purify these isomers with extremely similar polarities. Fig 4 shows a slight differentiation of isomer pairs of isoorientin/orientin-6″-laurate and isovitexin/vitexin-6″-laurate. Silica gel column chromatography has also been used in the purification of flavonoids and flavonoid esters. Afifi *et al*. separated isoorientin from *Arum palaestinum* by silica gel column chromatography [28], and Kontogianni *et al*. purified naringin esters by column chromatography on silica gel (their products were eluted with acetonitrile/methanol/water (8:2:0.3, v/v/v)) [29]. In our study, the TLC analyses were performed with a mixture of dichloromethane/diethyl ether/methanol (65:30:5, v/v/v) as eluents, and clear spots without overlapping were obtained (Fig 5). Accordingly, the isolation of AOB acylated derivatives was performed on a silica gel column using the same eluents (dichloromethane/diethyl ether/methanol, 80:15:5, v/v/v). The isolated products were reanalyzed by HPLC (Fig 6), which confirmed that adequate separation was achieved by silica gel column chromatography. From these results, it can be concluded that in comparison with the preparative HPLC method, silica gel column chromatography is more efficient, convenient, and economical for the separation of AOB acylated derivatives.

### Effects of AOB and acylated AOB on acrylamide formation in potato crisps

**Fig 7:**
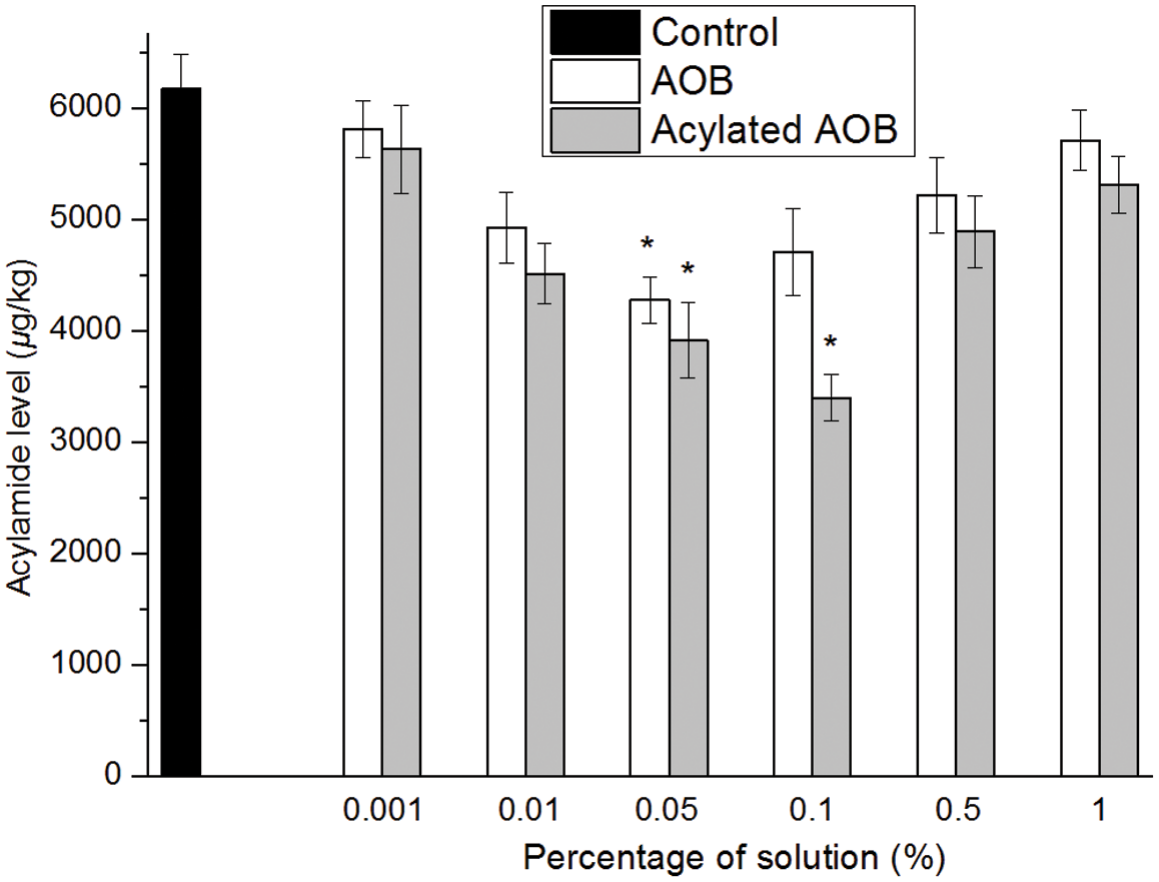
Relationship between acrylamide levels and different conditions of AOB and acylated AOB immersion treatments in potato crisps. Data values are mean ± SD (*n* = 3). Bars with an asterisk indicate significant difference from the control (*p* < 0.05).

Researchers have studied the effects of plant or fruit extracts containing flavonoids or phenolic compounds on acrylamide formation in model systems and in fried potato crisps with satisfactory results [30,31]. Fig 7 shows the relationships between acrylamide levels and different treatments of potato crisps. The results indicated that acylated AOB was more efficient for inhibiting acrylamide formation in fried potato crisps. Specifically, potato crisps with 0.001%, 0.01%, 0.05%, 0.1%, 0.5%, and 1% AOB treatments induced 5.8%, 20.2%, 30.7%, 23.6%, 15.4%, and 7.4% inhibition of acrylamide formation, respectively. In contrast, the levels of acrylamide of potato crisps with 0.001%, 0.01%, 0.05%, 0.1%, 0.5%, and 1% acylated AOB treatments were 8.8%, 26.9%, 36.5%, 44.9%, 20.7%, and 13.9% less than the control group, respectively. Statistical analyses showed that among the treatments, 0.05% AOB, and 0.05% and 0.1% acylated AOB groups significantly (*p* < 0.05) reduced the content of acrylamide levels in fried potato crisps. The enhanced potency of acylated AOB could be attributed to its higher solubility in a lipidic food system such as fried potato crisps. Acylated AOB may be more accessible to scavenge reactive carbonyls formed in Maillard reactions (which are responsible for the formation of acrylamide in food systems), and this property might result in reduced acrylamide levels [32]. It should be pointed out that results didn’t show significant concentration-dependent relationship in different ranges of treatments or even anti-concentration-dependent relations in high ranges of treatments. This reverse tendency may be attributed to “antioxidative paradox” which had been first reported in 1993 [33] and observed by different researchers since then [34, 35, 36].

## Conclusions

In conclusion, AOB can be successfully acylated with lauric acid as the acyl donor using *Candida antarctica* lipase B in *tert*-amyl-alcohol, with four types of purified acylated flavonoids obtained through column chromatography on silica gel. The addition of AOB and acylated AOB containing natural flavonoids and acylated flavonoids resulted in a reduction of acrylamide levels in fried potato crisps. Statistical analyses showed that the 0.05% AOB and 0.05% and 0.1% acylated AOB groups significantly (*p* < 0.05) reduced the content of acrylamide in potato crisps. These results indicate that the application of *Candida antarctica* lipase B might allow the total acylation of plant or fruit extracts rich in glycosylated flavonoids. Acylation may enhance glycosylated flavonoids’ inhibitory activity toward the formation of acrylamide in a lipidic food system. Further studies are needed to investigate the structures of acyl donors and immersion times on the formation of acrylamide in all potato-based products and to clarify the mechanism whereby acylated glycosylated flavonoids inhibit acrylamide.
